# Dietary Omega-3 Supplementation with Linseed and *Padina pavonica* Protects Rabbit Spermatozoa Against In Vitro LPS-Induced Damage

**DOI:** 10.3390/antiox15030289

**Published:** 2026-02-26

**Authors:** Alda Quattrone, Nour Elhouda Fehri, Olimpia Barbato, Majlind Sulçe, Cesare Castellini, Simona Mattioli, Enkeleda Ozuni, Daniele Vigo, Francesca Falcinelli, Livio Galosi, Lucia Biagini, Giacomo Rossi, Giovanni Ricci, Elena Moretti, Maria Laura Marongiu, Giulia Collodel, Gabriele Brecchia, Giulio Curone, Laura Menchetti

**Affiliations:** 1 Department of Veterinary Medicine and Animal Sciences, University of Milan, Via dell’Università 6, 26900 Lodi, Italy; alda.quattrone@unimi.it (A.Q.); nour.fehri@unimi.it (N.E.F.); daniele.vigo@unimi.it (D.V.); giulio.curone@unimi.it (G.C.); 2 Department of Veterinary Medicine, University of Perugia, Via San Costanzo 4, 06126 Perugia, Italy; francesca.falcinelli@collaboratori.unipg.it (F.F.); giovanni.ricci@unipg.it (G.R.); 3 Faculty of Veterinary Medicine, Agricultural University of Tirana, Kodër Kamëz, 1029 Tirana, Albania; msulce@ubt.edu.al (M.S.); enkelejda.ozuni@ubt.edu.al (E.O.); 4 Department of Agricultural, Food and Environmental Science, University of Perugia, Borgo XX Giugno 74, 06124 Perugia, Italy; cesare.castellini@unipg.it (C.C.); simona.mattioli@unipg.it (S.M.); 5 School of Biosciences and Veterinary Medicine, University of Camerino, Via Circonvallazione 93/95, 62024 Matelica, Italy; livio.galosi@unicam.it (L.G.); lucia.biagini@unicam.it (L.B.); giacomo.rossi@unicam.it (G.R.); laura.menchetti@unicam.it (L.M.); 6 Department of Molecular and Developmental Medicine, University of Siena, 53100 Siena, Italy; elena.moretti@unisi.it (E.M.); giulia.collodel@unisi.it (G.C.); 7 Department of Veterinary Medicine, University of Sassari, Via Vienna 2, 07100 Sassari, Italy; marongiu@uniss.it

**Keywords:** mele infertility, polyunsaturated fatty acids (PUFA), n-3 PUFA, lipopolysaccharide, rabbit fertility, sperm motility, TLR4, oxidative stress

## Abstract

Omega-3 polyunsaturated fatty acids (n-3 PUFAs) are recognized for their beneficial effects on male fertility. This study evaluated the protective effects of dietary n-3 PUFAs from extruded linseed, alone or combined with the alga *Padina pavonica*, against in vitro lipopolysaccharide (LPS)-induced sperm dysfunction in rabbits. Twelve bucks were fed for 60 days a control diet (CNT), a diet containing 5% extruded linseed (L), or 5% extruded linseed plus 0.2% *P. pavonica* extract (LPP). Ejaculates were exposed in vitro to increasing LPS concentrations (0, 400, and 600 µg/mL), and sperm motility was evaluated at 0, 1, 2, and 4 h using computer-assisted sperm analysis. LPS markedly impaired sperm motility in the CNT group, increasing the percentage of static spermatozoa (*p* < 0.001) and reducing sperm progressive motility (*p* < 0.001), with complete immobility observed at 600 µg/mL after 4 h. Conversely, sperm from L and LPP groups maintained significantly higher progressive motility, lower static sperm, and improved kinematic parameters throughout the LPS challenge (*p* < 0.05). Dietary n-3 PUFA supplementation also attenuated LPS-induced TLR4 activation and reduced lipid peroxidation, as indicated by lower seminal TBARS levels. No histological alterations were detected in the male reproductive tract. These findings indicate that n-3 PUFA supplementation, particularly linseed combined with algae, mitigates LPS-induced sperm dysfunction in vitro.

## 1. Introduction

Omega-3 polyunsaturated fatty acids (n-3 PUFAs) are widely recognized as key modulators of male reproductive function across mammalian species, owing to their fundamental roles in sperm membrane composition, redox homeostasis, and regulation of inflammatory pathways [1,2]. Adequate dietary intake of n-3 PUFAs has been consistently associated with improved semen quality, characterized by higher sperm concentration, enhanced motility, and more favorable sperm morphology in both humans and animal models, including rabbits [3,4,5]. Collectively, clinical and experimental evidence underscores the importance of targeted nutritional strategies in supporting male fertility and reproductive performance [5,6,7].

The beneficial effects of n-3 PUFAs on sperm function arise from multiple, interconnected mechanisms [8]. Structurally, fatty acids such as α-linolenic acid (ALA), eicosapentaenoic acid (EPA), and docosahexaenoic acid (DHA) are incorporated into the sperm plasma membranes, where they modulate membrane fluidity and stability, properties that are essential for sperm motility, capacitation, and the acrosome reaction [9]. Mammalian spermatozoa are indeed particularly enriched in PUFAs, which can account for up to 40–60% of total membrane fatty acids, with DHA and docosapentaenoic acid (DPA) typically predominating, although marked species-specific variation exists [10,11]. Beyond their structural role, n-3 PUFAs exert pronounced antioxidant and anti-inflammatory effects that are highly relevant for male fertility. In particular, EPA and DHA act as precursors of specialized pro-resolving lipid mediators, including resolvins and protectins, which actively suppress inflammatory signaling, reduce cytokine production, and promote resolution of inflammation [4]. Moreover, at the mitochondrial level, n-3 PUFA support mitochondrial bioenergetics by facilitating efficient ATP production required for flagellar activity, while concurrently limiting excessive generation of reactive oxygen species (ROS) [12]. Through these mechanisms, n-3 PUFAs contribute to the maintenance of redox balance and protect spermatozoa from inflammation-induced functional damage. Nevertheless, the high PUFA content of sperm membranes also renders spermatozoa particularly vulnerable to oxidative stress. Given their limited intrinsic antioxidant defenses, disruption of redox homeostasis can rapidly lead to lipid peroxidation, DNA damage, impaired motility, and reduced fertilizing capacity [13]. Accordingly, dietary strategies aimed at increasing the incorporation of n-3 PUFAs into sperm membranes, particularly when combined with antioxidant support, have been consistently associated with improved semen quality across mammalian species [14].

In this context, recent research in livestock production has increasingly focused on sustainable nutritional approaches capable of providing both n-3 PUFAs and bioactive antioxidant compounds. Among plant-based sources, extruded linseed represents one of the most widely used ingredients in animal nutrition and has demonstrated beneficial effects on reproductive performance in several species, including rabbits [15,16]. In parallel, marine algae have emerged as innovative and environmentally sustainable sources of long-chain n-3 PUFAs for animal feeding. Notably, the brown alga *Padina pavonica* is rich in EPA and DHA, as well as polyphenols, polysaccharides, carotenoids, and minerals, conferring antioxidant, anti-inflammatory, and cytoprotective properties [17,18,19].

Despite growing interest in nutritional interventions, male infertility remains a growing global concern and is increasingly recognized as a multifactorial condition influenced by genetic, endocrine, environmental, inflammatory, and lifestyle-related factors [20]. In both humans and intensively managed livestock systems, reproductive efficiency is frequently compromised by inflammatory insults to the reproductive tract [21]. Lipopolysaccharide (LPS), a major endotoxin of the outer membrane of Gram-negative bacteria, represents a potent inducer of inflammation and is widely used in experimental settings to investigate inflammation-associated dysfunctions [22]. Exposure to LPS has been shown to impair sperm motility, disrupt mitochondrial function, and promote oxidative damage, primarily through activation of Toll-like receptor 4 (TLR4). TLR4 signaling initiates pro-inflammatory cascades and enhances oxidative stress, leading to lipid peroxidation and loss of sperm functionality [22,23]. Rabbits represent a suitable experimental model in this context, as their reproductive physiology and responsiveness to both nutritional and inflammatory challenges closely resemble those observed in humans [24].

Based on this framework, we hypothesized that dietary supplementation with n-3 PUFAs, administered through extruded linseed alone or in combination with *Padina pavonica*, is able to protect rabbit spermatozoa from LPS-induced oxidative stress and inflammatory damage. We further hypothesized that these protective effects are mediated, at least in part, by modulation of TLR4 signaling and attenuation of membrane lipid peroxidation. Accordingly, the present study aimed to evaluate the anti-inflammatory and antioxidant effects of n-3 PUFAs on semen quality using an in vitro model of inflammation-induced sperm damage in rabbits. To achieve this objective, sperm motility and kinematic parameters were assessed following in vitro LPS challenge using computer-assisted sperm analysis, while TLR4 expression and lipid peroxidation were evaluated by immunofluorescence and thiobarbituric acid reactive substances (TBARS) assay, respectively. Finally, exploratory histopathological observations were performed on a limited subset of animals to confirm the healthy status of the reproductive organs.

## 2. Materials and Methods

### 2.1. Animals and Experimental Diets

The experimental trial was carried out at the experimental facilities of the Department of Agricultural, Food and Environmental Sciences, University of Perugia (Perugia, Italy), in compliance with European Union Directive 2010/63/EU regulating the use of animals for scientific purposes. All experimental procedures were designed to minimize animal distress and to limit the number of animals used, while ensuring adequate scientific validity. Animal health and welfare were routinely monitored throughout the study by the farm veterinarian.

A total of twelve New Zealand White rabbit bucks, 8 months of age and with a mean body weight of approximately 4 kg, were enrolled in the experiment. Animals were housed individually in standard cages under controlled environmental conditions, with ambient temperature maintained between 18 and 23 °C, relative humidity ranging from 60% to 75%, and a 16 h light/8 h dark photoperiod. Rabbits were randomly assigned to three experimental groups (*n* = 4 per group) and fed different pelleted diets formulated to meet the nutritional requirements of adult bucks [25]. The three experimental diets consisted of a control diet (CNT), based on a standard commercial pelleted feed; the same diet supplemented with 5% extruded linseed (L); and the same diet supplemented with 5% extruded linseed in combination with 0.2% *Padina pavonica* algae extract (LPP). All diets were formulated to be isoenergetic and isoproteic. Extruded linseed was included as a source of n-3 PUFAs, while *Padina pavonica* was added to provide additional very long-chain PUFAs and other bioactive compounds. The ingredient composition and chemical characteristics of the experimental diets are reported in Table 1 and in a previously published study [18]. The inclusion level of *Padina pavonica* extract was limited to 0.2% to preserve diet palatability and nutritional balance, considering the high ash content of the extract and previous reports of reduced feed intake at higher levels of algae supplementation [26,27]. The fatty acid methyl esters (FAMEs) of the experimental diets were determined as previously described [18]. Fatty acids were expressed as a percentage of total identified fatty acids, and the resulting fatty acid profiles are reported in Table 2.

All dietary treatments were administered for 60 days prior to semen collection and in vitro LPS challenge. This feeding period was selected to cover a complete spermatogenic cycle in rabbits, which lasts approximately 50 days, thereby allowing sufficient time for dietary n-3 PUFAs to be metabolized and potentially incorporated into sperm cell membranes [3].

### 2.2. Semen Collection and In Vitro LPS Challenge

Semen samples were collected from each buck using an artificial vagina in the presence of a teaser doe, following standard procedures routinely applied in rabbit reproduction [29]. Immediately after collection, ejaculates were subjected to a macroscopic evaluation, and only samples showing normal appearance and absence of visible contaminants were included in the study. Semen samples were then immediately transported to the laboratory and maintained at 37 °C until further processing. In the laboratory, ejaculate volume was recorded, and sperm concentration (×10^6^ spermatozoa/mL) was determined using established analytical methods [21]. Each ejaculate was subsequently divided into equal aliquots, each containing 5 × 10^6^ spermatozoa, and diluted at a ratio of 1:5 in modified Tyrode’s albumin lactate pyruvate (TALP) medium. Prior to dilution, both fresh semen and the TALP extender were equilibrated at the same temperature (37 °C), and mixing was performed under temperature-controlled conditions to avoid thermal shock. This experimental design was consistent with the protocol adopted in our previous investigations [21], ensuring methodological continuity while extending the scope of the research. For each aliquot, three technical replicates were prepared, resulting in a total of 432 samples.

Aliquots were incubated at 37 °C under humidified conditions with 5% CO_2_ and exposed to different concentrations of LPS from *Escherichia coli* O127:B8 (Sigma-Aldrich, Steffeld, Germany). Samples were treated with LPS at concentrations of 0 µg/mL (No LPS), 400 µg/mL, and 600 µg/mL. The same LPS strain and preparation previously employed in our earlier study were used to maintain experimental and methodological consistency [21]. The selected LPS concentrations were based on prior evidence demonstrating their ability to induce a marked and reproducible impairment of rabbit sperm motility in vitro [21]. Following LPS treatment, sperm motility and kinematic parameters were evaluated immediately after LPS exposure (0 h) and after 1, 2, and 4 h of incubation using a computer-assisted sperm analysis (CASA) system (ISAS^®^, model ISASv1; Proiser R+D S.L., Valencia, Spain) equipped with an HS640C high-speed video camera. The CASA system settings for motility parameters were the same as those established for rabbit semen by previous experiments [30]. Briefly, a 10 μL drop from each aliquot of semen was loaded onto a Makler chamber prewarmed to 37 °C. All analyses were conducted using a microscope equipped with a heated stage maintained at 37 °C. For each sample, a minimum of six microscopic fields were examined, resulting in the analysis of at least 300 sperm tracks. Semen samples were recorded at a frame rate of 100 Hz for 1 s, allowing the acquisition of 12–200 consecutive images per field [21]. The evaluated parameters included the percentage of static spermatozoa, progressive motility (%), non-progressive motility (%), curvilinear velocity (VCL, µm/s), straight-line velocity (VSL, µm/s), average path velocity (VAP, µm/s), linearity (LIN, %), straightness (STR, %), amplitude of lateral head displacement (ALH, µm), and beat-cross frequency (BCF, Hz) [29].

### 2.3. Immunofluorescence Staining for Toll-like Receptor 4 Detection in Rabbit Sperm 

Immunofluorescence staining was performed on fresh semen samples collected from each buck to investigate whether dietary n-3 PUFA supplementation could influence TLR4 expression in spermatozoa following the in vitro LPS challenge. Sperm samples were examined both in the absence of LPS and after incubation with LPS at a concentration of 400 µg/mL for 4 h. This concentration was selected as the minimum effective dose capable of inducing a marked impairment of rabbit sperm motility in vitro [21], while still preserving overall sperm structural integrity. For this reason, this concentration was considered suitable to reproduce a consistent inflammatory challenge and was used as the single representative condition for subsequent immunofluorescence analyses aimed at assessing LPS-induced TLR4 immunoreactivity.

Sperm smears were initially incubated for 20 min at room temperature in phosphate-buffered saline (PBS) containing 1% bovine serum albumin (BSA) and 5% normal goat serum (NGS) to block nonspecific antibody binding. Slides were then placed in a humidified chamber and incubated overnight at 4 °C with a monoclonal anti-TLR4 primary antibody (CD284, clone HTA125; eBioscience, Invitrogen, Thermo Fisher Scientific, Carlsbad, CA, USA) diluted 1:200. After washing, the primary antibody was detected using a goat anti-mouse secondary antibody conjugated with Alexa Fluor 488 (Invitrogen, Thermo Fisher Scientific), applied at a dilution of 1:500 for 1 h at room temperature. Negative control slides were processed in parallel by omitting the primary antibody. Nuclear counterstaining was performed using DAPI (Vysis, Downers Grove, IL, USA). Stained slides were examined using a Leica DMI 6000 fluorescence microscope (Leica Microsystems GmbH, Wetzlar, Germany), and images were acquired with the Leica AF6500(Leica Microsystems GmbH, Wetzlar, Germany) imaging system. For each sample, at least 200 spermatozoa were evaluated to ensure a representative assessment of TLR4 immunoreactivity.

### 2.4. Assessment of Lipid Peroxidation Through Thiobarbituric Acid Reactive Substances (TBARS) Assay

Lipid peroxidation was evaluated by quantifying malondialdehyde (MDA), a major secondary product of oxidative degradation, using the thiobarbituric acid reactive substances (TBARS) assay. In this method, MDA reacts with thiobarbituric acid (TBA) to form a colored MDA–TBA_2_ adduct, which can be quantified spectrophotometrically [31]. MDA levels, expressed as TBARS, were quantified using a multispecies colorimetric assay kit (Zx-44116-96, ZellBio GmbH, Berlin, Germany), following the manufacturer’s instructions. Absorbance was measured at 535 nm with a Tecan Infinite Pro 200 spectrophotometer (Tecan Trading AG, Männedorf, Switzerland). The analytical sensitivity was 0.36 μM, and the intra-assay and inter-assay coefficients were 3.5% and 4.5%, respectively.

### 2.5. Exploratory Histopathological Evaluation of the Male Reproductive Tract

Exploratory histopathological evaluations were performed on a limited subset of animals to confirm the absence of reproductive tract pathology. Given the restricted sample size, this analysis was qualitative in nature and was not intended to draw definitive conclusions regarding dietary effects on tissue morphology. Animals were euthanized by an overdose of sodium pentobarbital, and tissue samples were collected from the prostate, bulbourethral glands, ampulla of the ductus deferens, epididymis, and rete testis. Detailed methodological procedures are reported in the Supplementary Materials. Histological evaluations were performed by pathologists blinded to the dietary group allocation.

### 2.6. Statistical Analysis

The number of animals included in the study was determined by practical and methodological constraints, as the experimental protocol required a large number of in vitro replicate measurements per subject for computer-assisted sperm analysis (CASA), performed under strictly standardized and synchronized conditions. Overall, 432 semen samples were generated and analyzed (see Section 2.2). A post hoc power analysis was conducted to assess the adequacy of the sample size. As power estimation for complex hierarchical models is not supported by G*Power^®^ (version 3.1), the calculation was simplified using a repeated-measures ANOVA design with three groups and 36 repeated measurements per subject. Assuming a large effect size (f = 0.77) and a significance level of α = 0.05, the achieved statistical power was estimated at 80%.

Diagnostic plots were used to assess model assumptions. Static, LIN, and BCF data were transformed using ln(x + 1) prior to statistical analysis. Data were then analyzed using Generalized Estimating Equations (GEE), with replicate (*n* = 3), LPS concentration (3 levels: 0/No LPS, 400, and 600 μg/mL), and time (4 levels: TpostLPS, T1h, T2h, T4h) included as within-subject factors using an unstructured working correlation matrix. The GEE models evaluated the main effects of diet (3 levels: CNT, L, LPP), concentration, and their interaction, with time included as a covariate. Several candidate distributions were assessed, and the final choice was based on the Quasi-Likelihood under the Independence Model Criterion (QIC), resulting in the use of a normal distribution with identity link for static spermatozoa and BCF, and a Tweedie distribution with log link for non-progressive motility, progressive motility, VCL, LIN, and ALH. Multiple comparisons were performed using the LSD method to assess differences between the supplemented groups and the control group at each time point and LPS concentration. Due to the highly skewed distribution of the data, the effects of LPS concentration and group on TBARs were analyzed using the Kruskal–Wallis test.

Statistical analyses were performed with SPSS Statistics version 25 (IBM, SPSS Inc., Chicago, IL, USA). We defined *p* ≤ 0.05 as significant.

## 3. Results

### 3.1. Effect of LPS on Sperm Motility

The percentage of static spermatozoa was significantly influenced by all tested factors, including incubation time, LPS concentration, experimental group, and the group × dose interaction (*p* < 0.001 for all), providing strong evidence consistent with the large effect size anticipated in the study design. When other factors were held constant, the proportion of static spermatozoa increased progressively with both incubation time and increasing LPS concentrations. With respect to dietary treatment, the lowest estimated marginal mean was observed in the LPP group (20.3 ± 3.5%), followed by the L group (24.3 ± 3.0%), while the highest value was recorded in the CNT group (45.6 ± 4.1%). The significant interaction effect was primarily driven by differences among dietary groups under LPS challenge conditions. In the absence of LPS, the proportion of static spermatozoa remained stable at approximately 20% across all groups.

**Figure 1:**
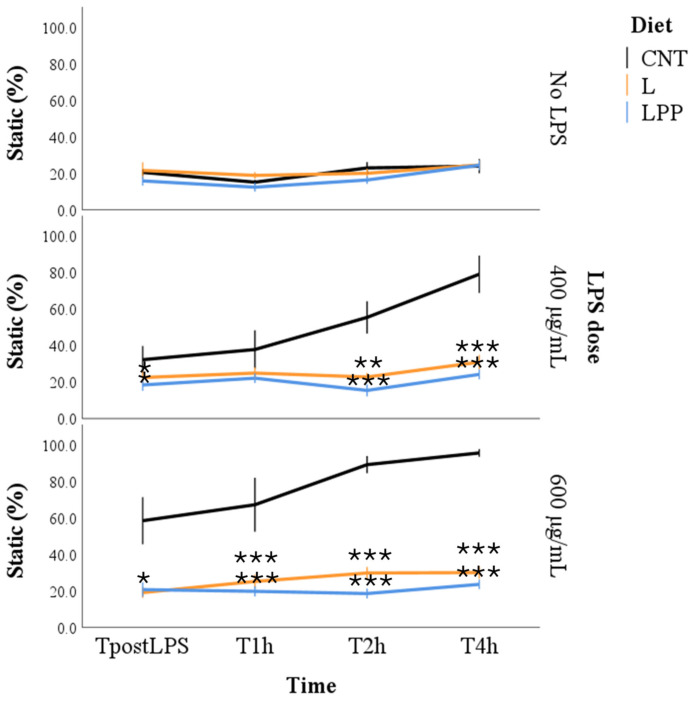
Percentage of static spermatozoa in semen samples from rabbits fed different experimental diets: control diet (CNT), diet supplemented with 5% extruded linseed (L), and diet supplemented with 5% extruded linseed plus 0.2% *Padina pavonica* extract (LPP). Sperm motility was assessed using a computer-assisted sperm analysis (CASA) system under basal conditions (0 µg/mL LPS, No LPS) and following in vitro exposure to lipopolysaccharide (LPS) at concentrations of 400 and 600 µg/mL. Measurements were taken immediately after LPS exposure (TpostLPS) and after 1 (T1h), 2 (T2h), and 4 (T4h) hours of incubation. Data are presented as mean ± standard error (SE). Asterisks indicate statistically significant differences compared with the control diet group (CNT) under the same LPS condition (* *p* < 0.05; ** *p* < 0.01; *** *p* < 0.001).

Following LPS exposure, a significant increase in static spermatozoa was detected in the CNT group as early as the immediate post-treatment time point (TpostLPS; *p* < 0.05). This effect intensified over time, reaching approximately 80% at 400 µg/mL and complete immobility (100%) at 600 µg/mL after 4 h of incubation (T4h). In contrast, sperm samples from the L and LPP groups maintained consistently low percentages of static spermatozoa (approximately 20%), regardless of LPS concentration or incubation time (Figure 1).

Progressive sperm motility was significantly influenced by dietary treatment (*p* < 0.001). The lowest estimated marginal mean was observed in the CNT group (16.6 ± 1.4%), which was significantly lower than that of the supplemented groups (*p* < 0.05). No significant differences were detected between the two supplemented groups, which exhibited comparable values (44.7 ± 3.0% for L and 47.6 ± 1.9% for LPP). Progressive motility was also negatively affected by increasing LPS concentrations (37.8 ± 1.6% in the absence of LPS, 36.2 ± 2.9% with 400 µg/mL, and 25.9 ± 3.7% with 600 µg/mL; *p* < 0.001), and a significant interaction between diet and LPS concentration was observed (*p* < 0.001).

**Figure 2:**
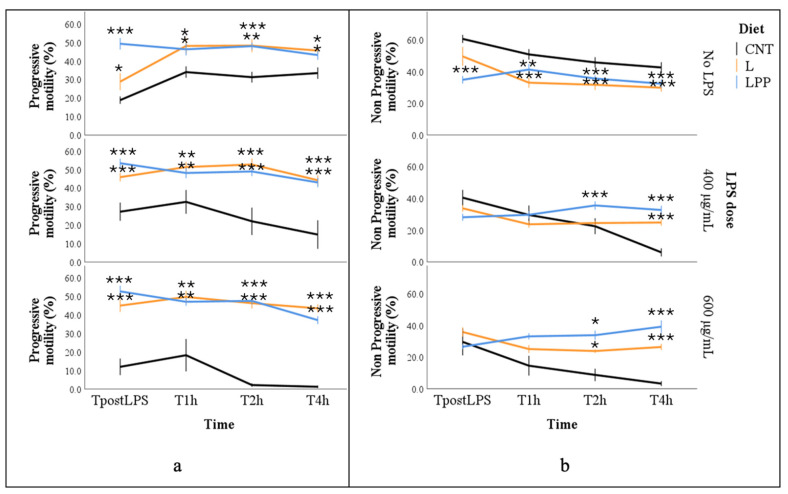
Percentage of progressive motility (panel (**a**)) and non-progressive motility (panel (**b**)) in semen samples from rabbits fed different experimental diets: control diet (CNT), control diet supplemented with 5% extruded linseed (L), and control diet supplemented with 5% extruded linseed plus 0.2% *Padina pavonica* extract (LPP). Sperm motility was evaluated using a computer-assisted sperm analysis (CASA) system under basal conditions (0 µg/mL LPS, No LPS) and after in vitro exposure to lipopolysaccharide (LPS) at concentrations of 400 and 600 µg/mL, immediately after LPS exposure (TpostLPS) and after 1, 2, and 4 h of incubation. Data are presented as mean ± standard error (SE). Asterisks indicate statistically significant differences compared with the control diet group (CNT) under the same LPS condition (* *p* < 0.05; ** *p* < 0.01; *** *p* < 0.001).

Across all incubation times and LPS concentrations, the two supplemented groups (L and LPP) consistently maintained higher progressive motility values, with mean percentages generally exceeding 30–40%, including after LPS exposure (*p* < 0.05). In contrast, in the absence of LPS (No LPS), CNT samples exhibited progressive motility values that never exceeded 40% and remained consistently lower than those observed in the L and LPP groups. Following LPS exposure, CNT samples showed a marked decline in progressive motility from T2 onward, ultimately resulting in complete loss of progressive motility (0%) at the highest LPS concentration (600 µg/mL; Figure 2a).

The percentage of spermatozoa exhibiting non-progressive motility was significantly affected by incubation time, LPS concentration, and the interaction between diet and LPS concentration (*p* < 0.001 for all effects). The lowest estimated marginal mean was observed at 400 µg/mL LPS, with values of 41.8 ± 2.8%, 18.0 ± 5.3%, and 28.5 ± 1.7% for the no LPS, 400 µg/mL, and 600 µg/mL conditions, respectively (*p* < 0.001). Independent of the other factors, non-progressive motility decreased over time (*p* < 0.001); however, the temporal pattern differed among dietary groups and LPS concentrations. In the absence of LPS, sperm samples from the L and LPP groups displayed lower percentages of non-progressive motility than those from the CNT group at most time points (*p* < 0.01), although values did not fall below 30%. After LPS exposure, group-related differences became more pronounced from T2 onward: spermatozoa from the two supplemented groups maintained non-progressive motility values between approximately 20% and 40%, whereas the CNT group exhibited a sharp decline, reaching values close to 0% four hours after LPS treatment at both tested concentrations (*p* < 0.01; Figure 2b).

**Figure 3:**
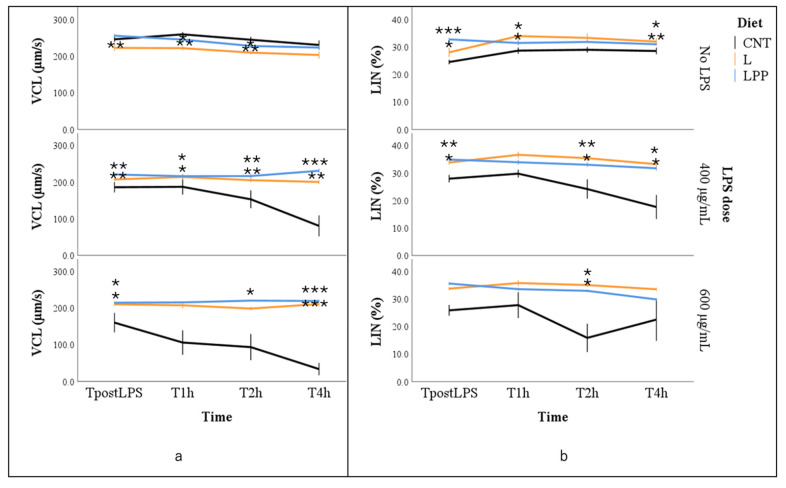
Curvilinear velocity (VCL, panel (**a**)) and linearity (LIN; panel (**b**)) in semen samples from rabbits fed different experimental diets: control diet (CNT), control diet supplemented with 5% extruded linseed (L), and control diet supplemented with 5% extruded linseed plus 0.2% *Padina pavonica* extract (LPP). Sperm motility was evaluated using a computer-assisted sperm analysis (CASA) system under basal conditions (0 µg/mL LPS, No LPS) and after in vitro exposure to lipopolysaccharide (LPS) at concentrations of 400 and 600 µg/mL, immediately after LPS exposure (TpostLPS) and after 1, 2, and 4 h of incubation. Asterisks indicate statistically significant differences compared with the control diet group (CNT) under the same LPS condition (* *p* < 0.05; ** *p* < 0.01; *** *p* < 0.001).

Curvilinear velocity (VCL) was significantly affected by all factors included in the model. Independent of dietary treatment, the regression coefficient (b) indicated a progressive reduction in VCL over incubation time (*p* < 0.05). Among dietary groups, the highest estimated marginal mean was observed in the LPP group, whereas no significant differences were detected between the CNT and L groups (174.4 ± 13.0, 203.6 ± 11.2, and 214.6 ± 3.1 µm/s for CNT, L, and LPP, respectively; *p* < 0.01). With respect to LPS exposure, the highest marginal mean VCL was recorded in the absence of LPS, followed by 600 and 400 µg/mL LPS (*p* < 0.001). In the absence of LPS, spermatozoa from the supplemented groups (L and LPP) occasionally showed slightly lower VCL values than those from the CNT group at 1 and 2 h of incubation (*p* < 0.05). However, mean values were comparable across groups and consistently exceeded 200 µm/s. The interaction effect was evident when comparing the response of the CNT group to LPS exposure. While spermatozoa from the L and LPP groups maintained VCL values of approximately 200 µm/s across incubation times and LPS concentrations, CNT samples exhibited a marked and rapid decline following LPS treatment, particularly at 600 µg/mL, reaching values markedly lower than those of the supplemented groups and approaching zero after 4 h of incubation (Figure 3a).

Linearity (LIN) was influenced exclusively by dietary treatment (*p* < 0.01). The estimated marginal mean for the CNT group was significantly lower than that observed in the supplemented groups (23.4 ± 2.4%, 33.4 ± 1.9%, and 32.7 ± 0.4% for CNT, L, and LPP, respectively; *p* < 0.001). Multiple comparisons confirmed that the CNT group consistently exhibited a lower percentage of linear spermatozoa, both in the absence and presence of LPS (*p* < 0.05; Figure 3b).

**Figure 4:**
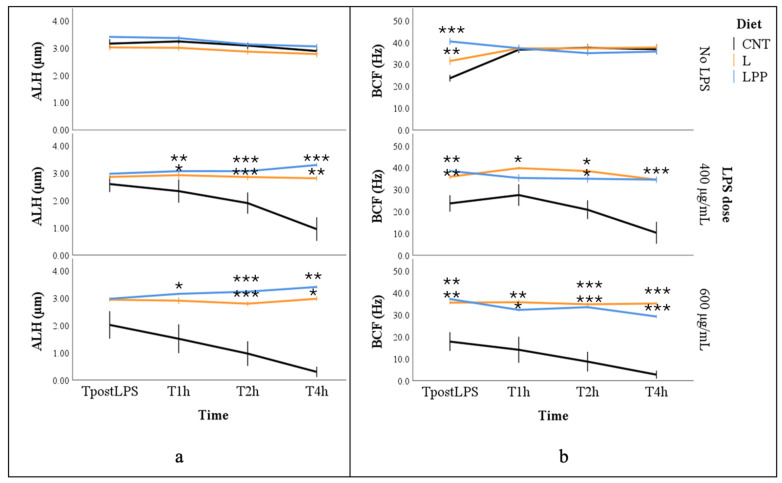
Amplitude of lateral head displacement (ALH, panel (**a**)) and beat cross frequency (BCF, panel (**b**)) in semen samples from rabbits fed different experimental diets: control diet (CNT), control diet supplemented with 5% extruded linseed (L), and control diet supplemented with 5% extruded linseed plus 0.2% *Padina pavonica* extract (LPP). Sperm motility was evaluated using a computer-assisted sperm analysis (CASA) system in samples not exposed to lipopolysaccharide (0 µg/mL LPS; No LPS) and after in vitro exposure to lipopolysaccharide (LPS) at concentrations of 400 and 600 µg/mL. Measurements were performed immediately after LPS addition (TpostLPS) and after 1, 2, and 4 h of incubation. Data are presented as mean ± standard error (SE). Asterisks indicate statistically significant differences compared with the control diet group (CNT) under the same LPS condition (* *p* < 0.05; ** *p* < 0.01; *** *p* < 0.001).

The amplitude of lateral head displacement (ALH) was significantly affected by LPS concentration (*p* < 0.001), with the highest marginal mean values observed in the absence of LPS and lower values at increasing LPS concentrations (3.2 ± 0.1, 2.2 ± 0.3, and 2.7 ± 0.1 µm for No LPS, 400 µg/mL, and 600 µg/mL, respectively). In addition, ALH decreased over time (*p* < 0.05), and a significant interaction between LPS concentration and dietary treatment was observed (*p* < 0.001). In samples not exposed to LPS, ALH values remained relatively stable over time, averaging approximately 3 µm in all dietary groups. After LPS exposure, spermatozoa from the CNT group showed a rapid and pronounced reduction in ALH, reaching values close to 1 µm at 400 µg/mL and approaching 0 µm at 600 µg/mL. In contrast, spermatozoa from the supplemented diet (L and LPP) groups maintained stable ALH values over time, remaining close to 3 µm even after LPS treatment, and were consistently higher than those observed in the CNT group (*p* < 0.05; Figure 4a).

Beat cross frequency (BCF) was significantly influenced by all experimental factors except incubation time (*p* < 0.001). The lowest marginal mean BCF values were recorded in the CNT group, whereas no significant differences were observed between the two supplemented groups (17.1 ± 3.4, 36.2 ± 1.1, and 35.0 ± 0.8 Hz for CNT, L, and LPP, respectively). Exposure to LPS significantly reduced BCF, with higher marginal mean values observed in samples not treated with LPS compared with those exposed to LPS (40.7 ± 1.8, 21.9 ± 6.1, and 24.2 ± 5.1 for No LPS, 400 µg/mL, and 600 µg/mL, respectively). Multiple comparisons showed that BCF values were significantly higher in the L and LPP groups, remaining between approximately 30 and 40 Hz even after LPS exposure (*p* < 0.05). In contrast, spermatozoa from the CNT group exhibited lower BCF values in the first evaluated samples (TpostLPS), averaging approximately 20 Hz both in the presence and absence of LPS. In LPS-treated samples, BCF values in the CNT group declined further over time, reaching approximately 10 Hz at 400 µg/mL and approaching 0 Hz at 600 µg/mL LPS (Figure 4b).

### 3.2. Expression of Toll-like Receptor 4 in Rabbit Sperm with Immunofluorescence Staining

**Figure 5:**
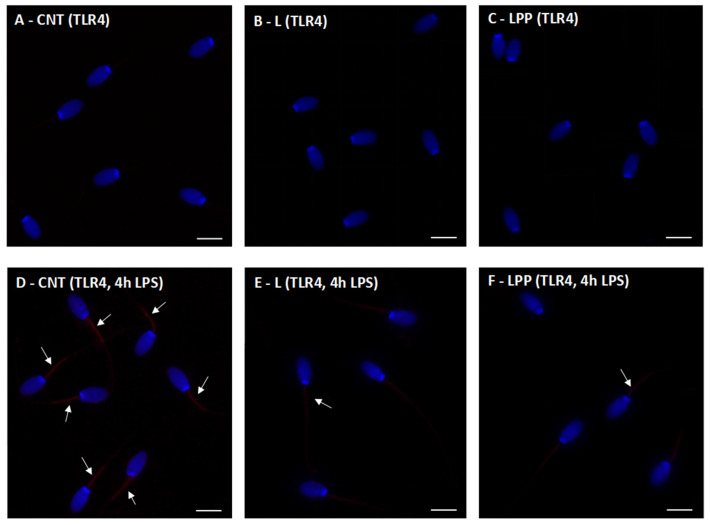
Ultraviolet (UV) micrographs of rabbit spermatozoa stained with an anti–Toll-like receptor 4 (TLR4) antibody. Panels (**A**–**C**) show spermatozoa from rabbits fed a control diet (CNT group; (**A**)), control diet supplemented with 5% extruded linseed (L group; (**B**)), and control diet supplemented with 5% extruded linseed plus 0.2% *Padina pavonica* algae extract (LPP group; (**C**)). Panels (**D**–**F**) show spermatozoa from the same dietary groups, CNT (**D**), L (**E**), and LPP (**F**), after 4 h of in vitro incubation with 400 µg/mL lipopolysaccharide (LPS). In panels (**A**–**C**), TLR4 immunofluorescence is weak and mainly localized to the midpiece of the sperm tail. Following LPS exposure, a stronger TLR4 signal is observed in spermatozoa from the CNT group ((**D**), red), whereas reduced fluorescence intensity is evident in the L (**E**) and LPP (**F**) groups. White arrows indicate representative spermatozoa showing TLR4-positive immunofluorescence. Sperm nuclei were counterstained with DAPI (4′,6-diamidino-2-phenylindole, blue). Scale bars = 5 µm.

Immunofluorescence staining with an anti–TLR4 antibody revealed a very weak signal in spermatozoa from rabbits fed the control diet (Figure 5A), the linseed-supplemented diet (Figure 5B), and the linseed plus algae-supplemented diet (Figure 5C). In all groups, TLR4 immunoreactivity was faint and primarily localized at the midpiece of the sperm tail.

After in vitro incubation with LPS for 4 h at a concentration of 400 µg/mL, TLR4 labeling markedly increased in spermatozoa from the control diet group, showing a strong and intense fluorescent signal (Figure 5D). In contrast, spermatozoa from rabbits fed the linseed (Figure 5E) and linseed plus algae (Figure 5F) diets exhibited a noticeably weaker TLR4 signal following LPS exposure.

### 3.3. Thiobarbituric Acid Reactive Substances (TBARS) Assay

**Figure 6:**
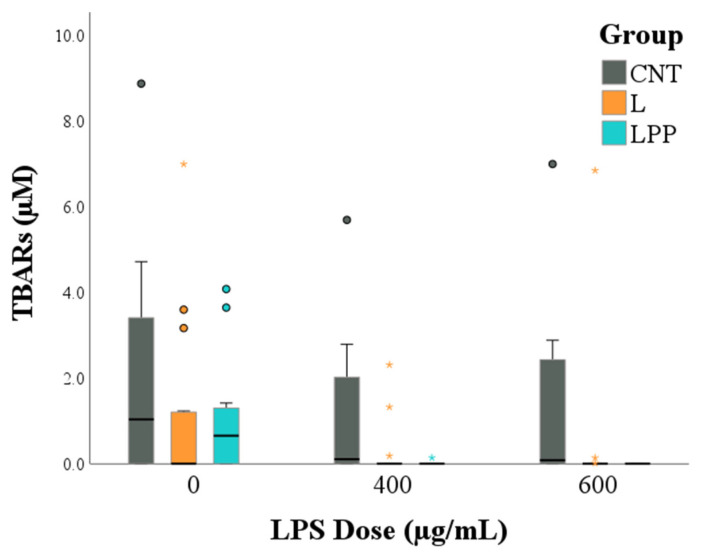
Thiobarbituric acid reactive substances (TBARS) levels in rabbit semen samples according to dietary group and lipopolysaccharide (LPS) concentration. Dietary groups were as follows: control diet (CNT), control diet supplemented with 5% extruded linseed (L), and control diet supplemented with 5% extruded linseed plus 0.2% *Padina pavonica* algae extract (LPP). Semen samples were evaluated in the absence of LPS (0 µg/mL) and after in vitro exposure to LPS at concentrations of 400 and 600 µg/mL. Circle symbols indicate values lying more than 1.5× the interquartile range below the first quartile or above the third quartile, while asterisks indicate extreme values lying more than 3.0× the interquartile range below the first quartile or above the third quartile.

Thiobarbituric acid reactive substances (TBARS) levels were significantly influenced by both dietary group and LPS concentration. Specifically, sperm samples from the control diet group (CNT) exhibited significantly higher TBARS values compared with those from the linseed-supplemented (L; *p* < 0.001) and linseed plus algae-supplemented (LPP; *p* = 0.001) groups. In addition, samples not exposed to LPS showed higher TBARS levels than samples treated with either 400 µg/mL (*p* = 0.002) or 600 µg/mL (*p* < 0.001) LPS (Figure 6).

### 3.4. Histological Analysis of the Male Rabbit Reproductive Tract

Histological examination of the reproductive tissues revealed a well-preserved physiological architecture across all experimental groups, with no pathological alterations detected. These qualitative observations indicate a normal reproductive tissue status and are reported in detail in the Supplementary Materials (Figure S1).

## 4. Discussion

The present study demonstrates that dietary n-3 PUFA supplementation, provided through extruded linseed alone or in combination with the brown alga *Padina pavonica*, positively modulates sperm motility in rabbits, with the most pronounced effects observed under inflammatory conditions. In particular, n-3 PUFA–enriched diets markedly protected spermatozoa from in vitro LPS-induced functional impairment, highlighting their relevance in counteracting inflammation-associated sperm dysfunction.

In LPS-untreated samples, rabbits receiving n-3 PUFAs–enriched diets exhibited significantly higher sperm progressive motility and linearity, confirming the established role of long-chain n-3 PUFAs in optimizing sperm kinematic parameters [32,33]. These effects are attributable to the incorporation of n-3 PUFAs into the sperm plasma membrane, which enhances membrane fluidity and flagellar dynamics, thereby providing the structural flexibility required for efficient propulsion and coordinated sperm movement [20]. Notably, the significant differences observed between the supplemented and control groups were not only present in the absence of LPS, but were also maintained immediately after the addition of the different LPS doses (i.e., at TpostLPS in samples treated with 400 or 600 µg/mL). This finding suggests that the mechanism of action of dietary supplementation may extend beyond anti-inflammatory protection. Given the lack of incubation time, these early alterations are unlikely to reflect a classical biological response to LPS and instead suggest an acute physicochemical stress induced by the LPS solution itself, such as osmotic imbalance or pH perturbation [34]. The preserved motility in the L and LPP groups at this time point indicates an enhanced intrinsic resilience, likely mediated by diet-induced modifications to the sperm membrane. Specifically, the incorporation of very long-chain n-3 PUFAs, such as DHA, increases membrane elasticity and structural integrity, thereby improving resistance to mechanical and osmotic shock [35]. Consequently, the sustained sperm motility observed in the supplemented groups throughout the subsequent incubation period likely reflects a dual protective mechanism: an initial structural resilience to the acute physicochemical insult of LPS, followed by the mitigation of the inflammatory and oxidative damage typically triggered by LPS.

As expected, in vitro LPS challenge induced a marked, dose- and time-dependent decline in sperm motility in control samples, culminating in complete immobility at the highest endotoxin concentration. This response aligns with a conserved pathogenic mechanism reported across species, including humans, whereby LPS activates TLR4-dependent inflammatory signaling, leading to excessive ROS generation, mitochondrial dysfunction, and lipid peroxidation, ultimately compromising sperm viability and motility [22,23,36,37,38]. In contrast, spermatozoa from rabbits fed n-3 PUFA–enriched diets largely preserved motility and kinematic parameters following LPS exposure. Specifically, the percentage of static spermatozoa remained stable and significantly lower than in controls, while progressive motility and other kinematic parameters, including VCL, LIN, ALH, and BCF, remained significantly higher and comparable to baseline values despite the LPS challenge. Notably, spermatozoa from the LPP group exhibited slightly superior kinematic performance, particularly in terms of curvilinear velocity and resistance to immobility. This enhanced functional preservation likely reflects a synergistic interaction between linseed-derived α-linolenic acid, which supports membrane remodeling and fluidity, and algae-derived bioactive compounds, including polyphenols, phlorotannins, and carotenoids, that provide complementary antioxidant protection [6,17,39]. Together, these components may stabilize PUFA-enriched membranes and enhance sperm resilience under endotoxin-induced stress [2,20].

Mechanistically, the preservation of sperm function in supplemented groups was closely associated with attenuated TLR4 activation and reduced lipid peroxidation [22]. In particular, increased TLR4 immunolabeling in control spermatozoa following LPS exposure suggests enhanced receptor activation, which may directly contribute to the reduction in sperm motility observed in this group. This association is likely mediated by the activation of pro-inflammatory pathways upon LPS-TLR4 binding, which triggers the production of ROS [23]. The resulting oxidative stress promotes lipid peroxidation of the sperm membrane and impairs mitochondrial function, leading to a decline in mitochondrial membrane potential [37]. Given that sperm motility is strictly dependent on ATP generated by the mitochondria in the midpiece, this TLR4-mediated cascade explains the observed reduction in motility in the control group. Conversely, sperm from n-3 PUFA–supplemented rabbits exhibited a reduced TLR4 immunoreactivity, suggesting modulation of receptor activation and/or downstream signal transduction. This modulatory effect is biologically relevant, as TLR4 activation is known to trigger NF-κB–dependent pro-inflammatory pathways, leading to increased production of cytokines such as TNF-α and IL-1β and amplification of oxidative stress [40,41,42,43]. By dampening this signaling pathway, n-3 PUFAs may therefore successfully interrupt the feed-forward loop between TLR4 activation and ROS generation, which is a recognized hallmark of endotoxin-induced male infertility [44,45,46]. Complementing these anti-inflammatory effects, decreased TBARS levels relative to control samples following LPS exposure were consistent with lower lipid peroxidation in both supplemented groups. Although membrane integrity was not directly assessed, reduced TBARS levels strongly suggest decreased peroxidative damage to sperm membranes or improved regulation of oxidative processes known to impair sperm motility and viability [47]. The tendency toward lower TBARS values at the highest LPS concentrations likely reflects reduced metabolic activity in severely immobilized spermatozoa, rather than a true attenuation of oxidative damage, and should therefore be interpreted with caution.

Although exploratory in nature due to the limited sample size, histological analyses did not reveal structural alterations in the male reproductive tract among the experimental groups under the conditions tested. While these observations do not allow definitive conclusions regarding tissue-level effects or dietary tolerability, they indicate that no major histopathological disruptions were evident at the time points examined. Future studies involving larger cohorts, extended supplementation periods, and quantitative histomorphometric analyses will be necessary to detect tissue changes, as well as to evaluate potential dose-dependent and long-term effects of n-3 PUFA supplementation on male reproductive tissues.

Finally, several limitations of the study should be acknowledged. First, sperm membrane fatty acid composition was not directly characterized; therefore, the degree and specificity of n-3 PUFA incorporation into sperm membranes remain to be determined. Moreover, key components of antioxidant and anti-inflammatory signaling pathways were not directly investigated. In particular, downstream mediators of TLR4 activation, such as NF-κB and pro-inflammatory cytokines (e.g., TNF-α and IL-1β), as well as enzymatic (superoxide dismutase, catalase, glutathione peroxidase) and non-enzymatic antioxidant defenses, were not quantified. Consequently, the mechanistic interpretation of the observed modulation of inflammatory and oxidative responses remains preliminary. Nonetheless, the convergence of functional, immunological, and biochemical evidence, including preserved sperm motility, reduced TLR4 immunoreactivity, and lower TBARS levels, strongly supports the involvement of coordinated anti-inflammatory and antioxidant mechanisms in the protective effects of dietary n-3 PUFA supplementation.

Future investigations should incorporate sperm membrane lipidomic analyses, detailed evaluation of inflammatory and redox signaling cascades, assessment of mitochondrial function, and comprehensive profiling of antioxidant defenses to further elucidate the molecular pathways underlying these effects and enhance their translational relevance.

## 5. Conclusions

Dietary supplementation with n-3 PUFAs in male rabbits improves sperm motility and mitigates the LPS-induced impairment of sperm function. These protective effects appear to involve the modulation of TLR4-related inflammatory pathways and the reduction in oxidative stress, as indicated by preserved motility parameters and decreased lipid peroxidation. The combination of extruded linseed and *Padina pavonica* proved particularly effective, suggesting a synergistic benefit between n-3 PUFAs and algae-derived antioxidants.

Overall, these findings support the use of targeted nutritional strategies to preserve sperm function under inflammatory conditions and to improve male reproductive performance in both experimental models and livestock production systems.

## Figures and Tables

**Table 1:** Ingredient composition of the experimental diets: control commercial pelleted feed (CNT), commercial feed supplemented with 5% extruded linseed (L), and commercial feed supplemented with 5% extruded linseed and 0.2% *Padina pavonica* algae extract (LPP).

Ingredients (%)	Diets		
	CNT	L	LPP
Wheat bran	25.07	24.87	24.90
Berley	13.33	13.00	13.00
Sunflower meal	12.00	11.67	11.50
Alfalfa meal	10.83	13.00	13.00
Sunflower husks	10.00	10.00	10.00
Beet pulp	7.50	5.66	5.50
Extruded linseed	-	5.00	5.00
Full- fat soybean	5.00	2.95	3.10
Wheat straw	4.17	2.00	2.00
Cane molasses	3.00	3.00	3.00
Wheat	2.50	2.50	2.50
Grape seed meal	2.33	1.67	1.70
Soybean hulls	0.00	0.16	1.70
Calcium carbonate	1.60	1.48	1.42
Soybean oil	0.78	-	-
Sodium chloride	0.40	0.40	0.40
Palm oil	0.33	-	-
Carboxymethylcellulose	0.30	0.30	0.30
Vitamin-mineral premix ^1^	0.25	0.25	0.25
Algae “*Padina pavonica*”	-	-	0.20
Lysine HCl	0.16	0.17	0.17
Liquid acidifier ^2^	0.15	0.15	0.15
Magnesium oxide	0.10	0.10	0.10
Methionine hydroxy analogue	0.06	0.07	0.07
Liquid choline	0.05	0.05	0.05
Vitamin E 50%	0.03	0.03	0.03
L Threonine	0.03	0.01	0.01
DL Methionine	0.03	-	-
Chemical composition (%)			
Crude protein	16.50	16.60	16.60
Ash	7.99	8.00	8.09
Crude fat	3.62	3.91	3.96
Fiber	17.16	16.82	16.91
Digestible Energy ^3^	2350	2350	2350

^1^ Vitamin Mineral premix composition per kg of diet: vitamin A 11,000 IU; vitamin D3 2000 IU; vitamin B1 2.5 mg; vitamin B2 4 mg; vitamin B6 1.25 mg; vitamin B12 0.01 mg; alpha-tocopherol acetate 50 mg; biotin 0.06 mg; vitamin K 2.5 mg; niacin 15 mg; folic acid 0.30 mg; D-pantothenic acid 10 mg; choline 600 mg; Mn 60 mg; Fe 50 mg; Zn 15 mg; I 0.5 mg; Co 0.5 mg. ^2^ Liquid acidifier composition: Formic acid 75%. ^3^ Digestible energy as kcal/kg, estimated following Maertens et al. [28].

**Table 2:** Fatty acids profile (% of total fatty acids) of the experimental diets (commercial feed (CNT), commercial feed integrated with 5% extruded linseed (L), and commercial feed integrated with 5% extruded linseed and 0.2% algae *Padina pavonica* extract (LPP)).

	CNT Diet	L Diet	LPP Diet
C14:0	0.18	0.20	0.20
C15:0	0.09	0.08	0.08
C16:0	13.92	11.14	11.16
C16:1 cis-9	0.18	0.20	0.18
C17:0	0.12	0.11	0.10
C17:1	0.05	0.05	0.05
C18:0	3.07	3.82	3.48
C18:1	25.39	23.89	23.98
C18:2 cis n-6, LA ^1^	47.63	32.41	33.53
C18:3 n-6, γ-ALA ^2^	0.21	0.12	0.19
C18:3 n-3, α-ALA ^2^	6.53	23.25	22.63
C20:0	0.28	0.22	0.22
C20:2	0.00	0.00	0.04
C20:3 n-3	0.00	0.00	0.02
C20:4 n-6, AA ^3^	0.00	0.00	0.08
C20:5 n-3, EPA ^4^	0.00	0.00	0.13
C22:0	0.00	0.00	0.23
C22:1	0.00	0.00	0.04
C22:5 n-3, DPA ^5^	0.00	0.00	0.05
C22:6 n-3, DHA ^6^	0.00	0.00	0.06
C24:1	0.00	0.00	0.03
Σ n-6 PUFA (%)	47.84	32.53	33.80
Σ n-3 PUFA (%)	6.53	23.25	22.89
n-6/n-3 Ratio	7.33	1.40	1.48
Total	97.65	95.49	96.48
Others	2.35	4.51	3.52

^1^ LA: linoleic acid; ^2^ ALA: α-linolenic acid; ^3^ AA: arachidonic acid; ^4^ EPA: eicosapentaenoic acid; ^5^ DPA: docosapentaenoic acid; ^6^ DHA: docosahexaenoic acid; Σ: sum of the individual fatty acids belonging to the indicated family (n-6 or n-3 polyunsaturated fatty acids).

## Data Availability

The original contributions presented in this study are included in the article/Supplementary Materials. Further inquiries can be directed to the corresponding authors.

## Supplementary Materials

The following supporting information can be downloaded at https://www.mdpi.com/article/10.3390/antiox15030289/s1. Figure S1: Representative histological sections of the testis (A–C) and epididymis (D–F) in the control group (CNT) and in the groups supplemented with 5% extruded linseed (L) and with 5% extruded linseed plus 0.2% *Padina pavonica* extract (LPP).
