# Supplementary material for: Dietary Omega-3 Supplementation with Linseed and Padina pavonica Protects Rabbit Spermatozoa Against In Vitro LPS-Induced Damage
Source: Antioxidants (Basel). 2026 Feb 26;15(3):289. doi: 10.3390/antiox15030289 (PMC13024459; doi:10.3390/antiox15030289)
Supplement: Supplementary file 1 [file antioxidants-15-00289-s001.zip › antioxidants-4133319-supplementary.pdf]

Supplementary Materials

Exploratory Histopathological Evaluation of the Male Reproductive Tract

To confirm the absence of evident pathological alterations in the male reproductive tract, an exploratory histopathological evaluation was performed on a limited subset of animals (n = 2 per group).

Samples were fixed in 10% neutral buffered formalin for 24 h and routinely processed for histological examination. Paraffin-embedded tissues were sectioned at 3 µm, mounted on Superfrost Plus slides (Histoline, Milan, Italy), dewaxed, and stained with hematoxylin and eosin (H&E) for microscopic evaluation. All histological assessments were performed by pathologists blinded to dietary group allocation.

Samples of examined organs displayed normal morphological features consistent with a healthy reproductive status, with no evidence of histopathological alterations (Figure S1).

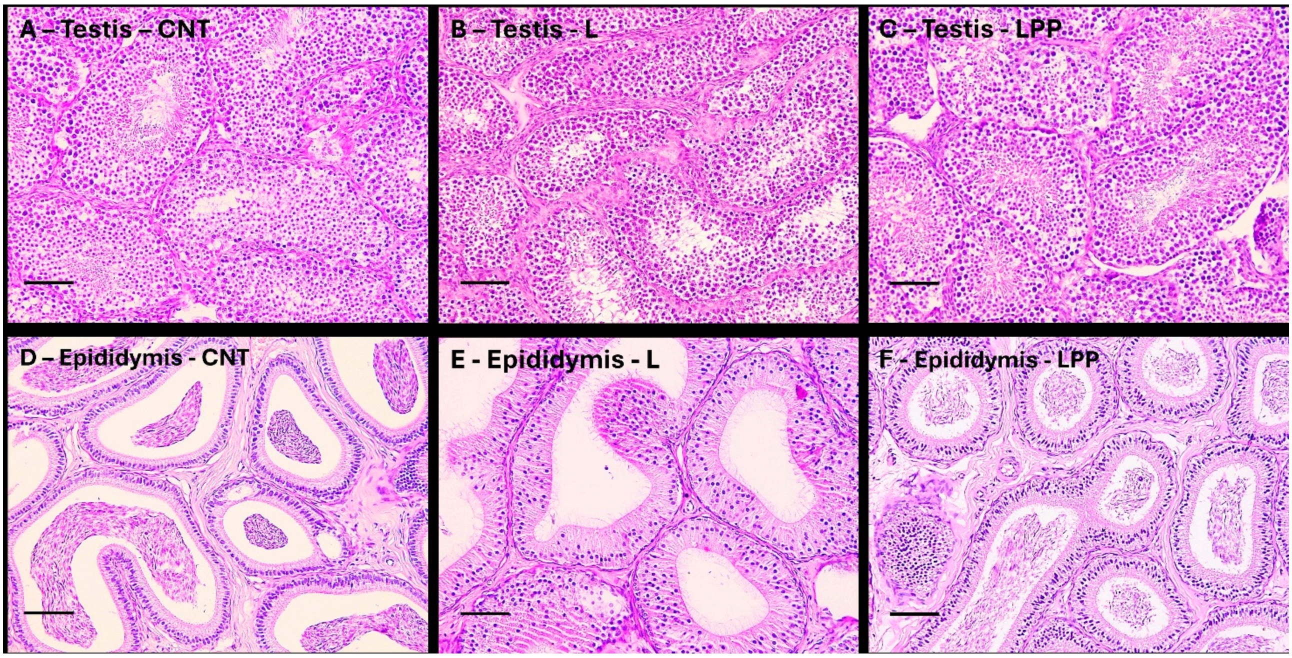

Figure S1. Representative histological sections of the testis (A-C) and epididymis (D-F) in the control group (CNT) and in the groups supplemented with 5% extruded linseed (L) and with 5% extruded linseed plus 0.2% *Padina pavonica* extract (LPP). Top panels (A–C) display the testicular parenchyma from CNT (A), L (B), and LPP (C) groups, all of which exhibit well-organized seminiferous tubules characterized by a complete germinal epithelium, active spermatogenesis, and well-defined interstitial compartments. Bottom panels (D–F) show the epididymal duct from CNT (D), L (E), and LPP (F) groups, highlighting preserved ductal structures with a continuous pseudostratified columnar epithelium, distinct apical stereocilia, and luminal spaces densely populated with spermatozoa. Across all experimental groups, there were no observable signs of oxidative stress-mediated damage, such as epithelial vacuolization, germ cell desquamation, or inflammatory infiltration, indicating the maintenance of physiological tissue integrity. Staining: Hematoxylin and Eosin (H&E); Scale bars = 100 µm.
